# Analyzing resilience influencing factors in the prefabricated building supply chain based on SEM-SD methodology

**DOI:** 10.1038/s41598-024-65271-2

**Published:** 2024-07-29

**Authors:** Mingchao Lin, Yifei Ren, Chao Feng, Xiaojuan Li

**Affiliations:** College of Transportation and Civil Engineering, Fujian Agriculture and Forestry University, Fuzhou, 350108 Fujian China

**Keywords:** Prefabricated building supply chain, Resilience, Influencing factors, Structural equation model, System dynamics, Civil engineering, Sustainability

## Abstract

The supply chain for prefabricated buildings (PB) currently grapples with pressing challenges. In order to ensure the safe and stable development of the prefabricated building supply chains (PBSC), this study aims to identify the key factors and internal mechanisms affecting the PBSC, and propose a supply chain resilience enhancement mechanism, so as to promote the sustainable development of the PB industry. The study combined a literature review and survey data to identify key resilience factors in PBSC. A Structural Equation Model (SEM) was used to explore the relationships between these factors. System dynamics were applied to create a simulation model, assessing the resilience impact level and conducting sensitivity analysis. The results show that the transportation and procurement processes are the most significant factors influencing supply chain resilience. The external environmental factors wielded a more pronounced impact on the overall evaluation of supply chain resilience than the delivery and use processes, but delivery and use processes are more sensitive. The study uses the Pressure-State-Response (PSR) model to suggest strategies for enhancing supply chain resilience. This study contributes to more sustainable and efficient construction practices by offering an innovative theoretical framework to analyze the factors influencing PBSC resilience and proposing enhancement strategies.

## Introduction

The global construction sector represents a significant contributor to greenhouse gas (GHG) emissions, estimated to contribute nearly 40% of the total emissions. This statistic primarily stems from the industry’s substantial energy consumption and carbon footprint, exacerbating the global challenge of climate change^1,2^. Recently, advancements in sustainable engineering and eco-friendly building technologies have led to the increasing prevalence of prefabricated buildings (PB), attributed to their efficiency, energy-saving capabilities, emission reduction, and resource efficiency^3,4^. Driven by policies and market guidance, prefabricated construction has begun to take shape within the industry chain. However, as the industry expands nationwide, the growing mismatch between management methods and production processes has become evident, leading to issues such as management disconnection. PB projects, as a product of the deep integration between construction and manufacturing industries, can leverage manufacturing experience and the supply chain as a fundamental unit to address overall management bottlenecks, such as logistics and fund flows during project construction^5^. Nevertheless, the successful execution of PB projects heavily relies on the stability and continuity of their supply chains^6^.

The prefabricated building supply chain (PBSC) spans various stages, including procurement, manufacturing, transportation, and installation, necessitating close collaboration among multiple supply chain participants^7^. These characteristics contribute to the supply chain facing several challenges. These challenges encompass frequent cost fluctuations, outdated technology, inadequate supply chain collaboration, and responsiveness to market fluctuations^8,9^. Particularly in light of unpredictable factors like natural disasters and economic fluctuations, the resilience of the supply chain emerges as a critical factor in ensuring project continuity^10^. To mitigate risks, the supply chain must be multidimensional and interdisciplinary, integrating event preparedness, providing efficient and effective responses, and returning to or enhancing the original state after an interruption^11^. Currently, research indicates that the PBSC in China is facing obstacles such as lack of coherence and slow progress^12^. Additionally, global supply chains are experiencing frequent disruptions and delays, posing barriers to resilience^13^. These challenges underscore the urgent need to enhance the resilience of the PBSC to foster the industry’s sustainable growth.

Research on PBSC has made some progress, but the exploration of supply chain resilience and its influencing factors is still in its infancy^5^. Existing studies have predominantly focused on generic strategies and approaches to supply chain management, while lacking in-depth analysis of the challenges and demands specific to PBSC^14^. Although some studies have attempted to identify factors that influence supply chain resilience, a nuanced understanding of how these factors impact supply chain performance in the PB sector remains limited. Moreover, there is insufficient research on methods and strategies to assess and enhance the resilience of PBSC, leaving significant room for further investigation. Therefore, there is a pressing need for more in-depth and systematic research to provide more reliable and effective theoretical and practical support for the enhancement of PBSC resilience^15^.

To ascertain the pivotal factors and inherent mechanisms impacting the PBSC, this study commenced by identifying resilience-influencing factors from the literature, subsequently confirming these key determinants. Next, a structural equation model was devised, encompassing six dimensions, to dissect the intricate interplay among various components of supply chain resilience. This model was then subjected to a simulation via system dynamics. Concludingly, employing the "pressure-state-response" (PSR) framework, this study deliberated strategies to enhance the safety resilience of the PBSC. This study contributes to systematically revealing the key resilience factors of PBSC and their dynamic changes through innovative and comprehensive methods and in-depth analyses. It also proposes enhancement strategies of practical significance, providing comprehensive theoretical and practical support for supply chain management.

## Literature review

### Research on prefabricated building supply chain management

In the 1990s, Koskela integrated the principles of supply chain management into the construction domain, establishing the foundational supply chain management framework for this sector^16^. The PBSC comprises a holistic process from manufacturing and transportation to the installation of prefabricated components used in construction endeavors. This complex system involves a myriad of stakeholders—ranging from suppliers and manufacturers to carriers and construction firms—all collaboratively ensuring the prompt delivery and integration of prefabricated modules^17^. Extensive studies have been undertaken on supply chain management within PB, examining the subject from diverse perspectives. Utilizing economic cost–benefit methodologies, scholars have identified pivotal areas to economize supply chain expenditures^18,19^. Furthermore, initiatives centered on risk management in the PBSC emphasize preemptive risk mitigation strategies, bolstering supply chain entities’ capacity to adeptly manage uncertainties^20,21^.

### The theory of resilience in prefabricated building supply chains

Supply chain resilience is a crucial aspect of supply chain management, focusing on how supply chains recover and maintain operations amidst unexpected events. Christopher and Peck^22^ defined supply chain resilience as the ability of a supply chain to cope with and recover from unexpected disruptions, emphasizing the importance of flexibility and redundancy. Subsequent studies have broadened this definition to include not only resilience but also the capacity to anticipate potential risks and prepare appropriate countermeasures^23^. Ponomarov and Holcomb^24^ further stated that supply chain resilience encompasses a system’s overall ability to prevent, adapt to, and recover from disruptions. Barroso et al.^25^ also explained that supply chain resilience requires supply chain members to effectively manage the negative impacts of disruptions to achieve common goals. Additionally, studies by Mohamed et al.^26^ and Yahaya and Qiping^27^ indicate that supply chain resilience involves preparing for unexpected events, responding to disruptions, and restoring supply chain structure and function through continuity strategies.

Supply chain resilience is centered on prevention, response, and recovery. Prevention involves mitigating potential supply chain disruptions through risk assessment and management. Real-time monitoring and prediction of supply chain risks using big data and machine learning techniques have become key tools for enhancing supply chain resilience^28^. Response involves implementing effective coping strategies when disruptions occur. Digital tools and real-time data analytics accelerate problem identification and resolution, thereby reducing the impact of disruptions^29^. Recovery focuses on returning to normal operations as quickly as possible after a disruption. Faruquee et al.^30^ suggest that the integrated use of internal and external resources, including digital transformation and partner networks, can effectively accelerate the recovery process. These core stages of resilience provide practical guidance for developing strategies to enhance supply chain resilience.

In the field of PB, supply chain resilience is particularly critical due to its unique material prefabrication and modularity characteristics. Prefabricated construction improves building efficiency and quality, and reduces construction time and cost by pre-fabricating building components in a factory environment and transporting them to the construction site for rapid assembly^4^. However, this approach is highly dependent on the coordination and optimization of all parts of the supply chain, and disruptions at any point can have a serious impact on the overall project^10^. Doloi^31^ highlights the need to improve supply chain resilience in PB projects by strengthening supplier management and optimizing logistics strategies. Therefore, understanding and improving the resilience of the PBSC requires focusing on the entire process from raw material procurement to final product delivery^32^, with influencing factors at each stage significantly impacting the stability and efficiency of the supply chain.

Strategic procurement planning and cost management are crucial for maintaining supply chain stability and competitiveness^33^. In the transportation stage, ensuring timely and safe delivery of materials to production facilities is essential for an uninterrupted supply chain. Partnering with a reliable transportation company can effectively reduce delays and costs^34^. At the production facility, optimizing project management and design accuracy ensures smooth production activities, enhancing adaptability and resilience^35^. The manufacturing stage focuses on cost control and the application of advanced production technologies and quality control systems, which improve efficiency, quality, and resilience to external shocks^36^. In the delivery and usage stage, optimizing supplier selection and enhancing maintenance and service processes improve responsiveness to customer needs and service quality, reinforcing customer-oriented resilience^37^. External environmental factors, such as changes in market demand and updates in laws and policies, necessitate continuous optimization of supply chain flexibility and adaptability to cope with external risks, ensuring continuity and efficiency^38^. Effective management of the entire supply chain relies on the coordination and optimization of these stages to support the long-term competitiveness and market responsiveness of the enterprise. The above discussion on the whole process resilience factors in the PBSC provides a theoretical reference for the construction of the SEM model in this study.

### Exploring the resilience of the prefabricated building supply chain

The resilience of PBSC is defined as the ability to ensure uninterrupted operation or rapid recovery to the original state or even better in the face of sudden shocks^6,39^. Azadegan et al.^40^ have proposed a framework that elucidates the organizational characteristics underpinning supply chain resilience, which include inherent resilience, anticipated resilience, and adaptive resilience. The determinants that influence supply chain resilience are multifaceted. As an illustration, Zhu et al.^41^ have probed into the PBSC under the EPC model, analyzing 17 crucial components integral to its resilience. Shishodia et al.^42^ utilized the SLNA dynamic literature review method to analyze the impact of information sharing on enhancing supply chain resilience. Wieland et al.^43^ collected data from surveys conducted at seven global companies to gather 14 factors influencing interruptions in the PB supply chain, and highlighted the relationship between supply chain resilience and improved supply chain performance. In today’s digitized era, the degree of information sharing within the supply chain plays a pivotal role in determining its resilience^44,45^. Regarding the assessment of supply chain resilience, some academics have introduced both methodological and ethnographic evaluation paradigms, as well as resilience assessment models, assisting managers in identifying key inflection points in the chain^11,46^. In conclusion, studies focusing on the resilience of the PBSC are still emergent. For fostering a robust foundation for this supply chain, more comprehensive research is of paramount importance.

Digital transformation offers new methods to control supply chain risks by optimizing risk management processes^47^. Consequently, PBSC companies are enhancing their adaptability to future challenges through the integration of advanced information technologies and the optimization of supply chain management strategies^48^. Building Information Modeling (BIM) technology improves the overall resilience of the PBSC by facilitating information integration and communication among project participants, thereby increasing transparency and collaboration efficiency^49^. Artificial intelligence (AI) technologies, particularly fuzzy logic, machine learning, and agent-based systems, have proven to be essential for enhancing supply chain resilience, helping organizations respond to rapidly changing market conditions^50^. During the global pandemic, the application of emerging IT capabilities, such as AI and blockchain, reduced supply chain vulnerabilities, increased resilience, and promoted sustainable business growth^51^. Additionally, research in the Indian construction industry has found that supply chain agility, resilience, and IT capabilities are critical for improving cost efficiency and delivery performance^52^. Studies on how logistics service providers utilize episodic adaptation theory to enhance supply chain resilience provide new insights into coping with high-impact, low-probability disruptive events by repurposing existing functions^53^. In conclusion, research on supply chain resilience is still in its infancy, and more comprehensive studies are essential to establish a solid foundation for this field.

### Advancing research methodologies in prefabricated building supply chain resilience

While prior studies have yielded insights into the model construction of PBSC management and contractual relationships^54^, researchers have also employed methodologies such as document network analysis^42^, the analytic hierarchy process, and fuzzy comprehensive evaluation^55^. However, traditional research approaches emphasize descriptive analysis and rely on simplistic models, lacking in complexity and dynamic analysis. To delve deeper into the crucial determinants influencing the resilience of PBSC, this study leverages the structural equation modeling (SEM) and system dynamics for evaluating and simulating the hypothetical model associated with resilience of the PBSC. SEM has been extensively adopted across disciplines such as social sciences and management. Its strength lies in estimating multiple equation systems simultaneously and addressing both observed and latent variables, circumventing the constraints of traditional multiple regression analyses that struggle with unobservable variables^56^. Various scholars have harnessed SEM to explore facets like the advancement of PB^57^, safety risk management^58^, and carbon emission abatement strategies^59^. System dynamics, introduced by Professor Forrester in the US, offers a quantitative means of examining intricate socio-economic systems, grounded in feedback control theory and utilizing computer-based dynamic simulation^60^. Scholars have employed system dynamics models, approaching subjects like construction waste management^61^, dynamic scenario assessments of carbon emission reduction in PBSC^62^, and progress management within PB projects^63^.

In summary, although supply chain resilience has been extensively analyzed in existing literature, many studies have clarified the role of technological innovations and management strategies in enhancing resilience. However, there remains a significant research gap in systematically evaluating the factors influencing the resilience of prefabricated supply networks. Additionally, while research on construction supply chain resilience is increasing, it primarily focuses on conventional buildings rather than the specific case of prefabrication^64,65^. Table 1 presents a comparative analysis of this study with existing research on the resilience of PBSC. This study aims to fill the research gap regarding the intricate and interrelated determinants affecting PBSC resilience by combining dynamic and static perspectives to provide a more comprehensive framework for PBSC resilience management. SEM offers an analysis of static causal relationships, while SD provides a dynamic perspective, revealing the inherent mechanisms of supply chain resilience. Augmented by the PSR model, it fills the gaps in strategies for enhancing supply chain resilience, thereby facilitating sustainable development and competitive enhancement in the industry.

**Table 1:** Comparison of current study with existing literature. This table delineates the advancements in research concerning the resilience of supply chains in prefabricated construction. It compares various studies by presenting their methodology, key findings, and the unique contributions of each study. This comprehensive overview assists in understanding the evolution of theoretical and practical approaches to enhancing resilience in prefabricated building supply chains over recent years.

References	Year	Methodology	Key findings	Contribution of current study
^6^	2021	Structural equation model (SEM)	A conceptual model of prefabricated building supply chain resilience was constructed, and the moderating roles of information factors and partnership factors were verified	This study further extends the SEM model by incorporating a system dynamics (SD) approach and adding a dynamic perspective to propose a more comprehensive SEM-SD assessment model of the factors influencing the resilience of the prefabricated building supply chain
^66^	2023	Structural equation model	The influencing factors of prefabricated building supply chain resilience were identified based on dynamic capacity theory, and critical paths were subsequently proposed	
^48^	2024	Structural equation model	The effects of component production, construction, and information uncertainty on prefabricated building project delays were quantified	
^45^	2023	Fuzzy set theory and system dynamics model	Key factors influencing prefabricated building supply chain resilience were identified, and the mechanisms by which these factors operate were explored	This study extends the SD model by integrating it with the static analysis from the previous SEM model, proposing a more comprehensive SEM-SD assessment model for the factors influencing the resilience of prefabricated building supply chains
^67^	2023	AHP and System dynamics model	Key metrics of prefabricated building supply chain resilience were identified, and a five-year resilience simulation was conducted	
^68^	2022	DEMATEL-ISM methodology and case studies	A prefabricated building supply chain resilience assessment model was developed, and the influencing factors at various levels were identified	This study not only identifies the factors influencing resilience but also extends the application of the PSR model. Based on the PSR model, targeted resilience enhancement strategies are proposed, providing a complete framework from identification to problem-solving
^11^	2023	AHP-DEMATEL and ISM models	A prefabricated construction supply chain risk impact indicator system was established, and key supply chain risk factors were identified	
^69^	2023	Pressure-state-response (PSR) modeling and case studies	A prefabricated construction supply chain supplier vulnerability assessment method was proposed, and its effectiveness was verified	

## Methods

**Figure 1:**
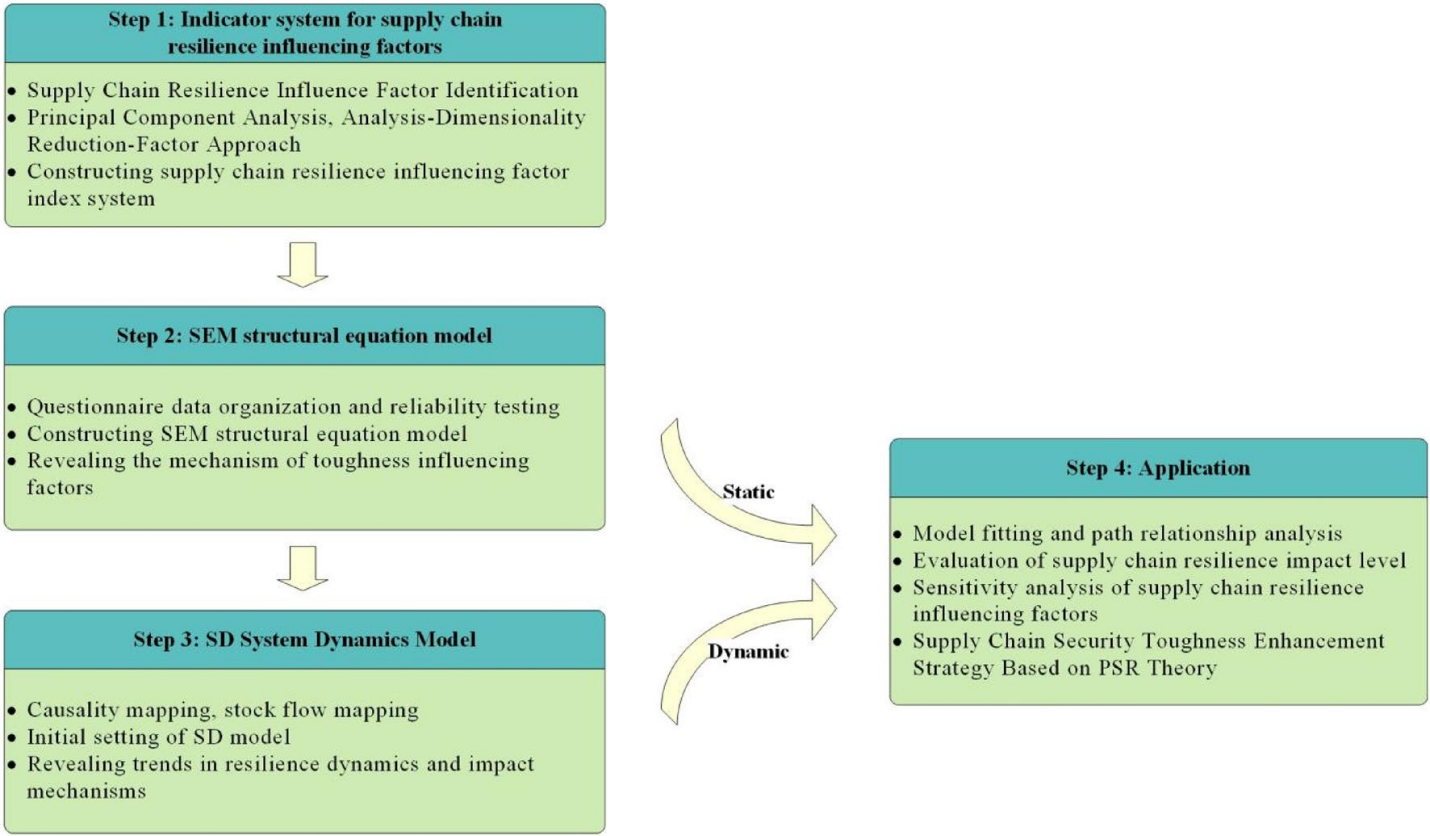
Research framework and process. This diagram presents a four-step approach to understanding and enhancing supply chain resilience in the prefabricated building industry. Step 1 establishes an indicator system to pinpoint influencing factors of resilience. Step 2 involves constructing and validating a Structural Equation Model (SEM) to analyze the identified factors. Step 3 develops a System Dynamics Model (SD) to track and project the behavior of these factors over time. The final step, Application, applies the findings to propose practical strategies for strengthening resilience, guided by the Pressure-State-Response (PSR) theory. The framework transitions from static identification to dynamic modeling and practical application, reflecting an integrated process for strategic resilience enhancement.

This investigation introduces an exploratory research framework. Initially, by leveraging both literary sources and a questionnaire survey, an index system for the factors affecting the resilience of the PBSC was delineated. Subsequently, the structural equation model was employed to probe the mechanisms underpinning these influencing factors and discern patterns of resilience. In the final phase, the system dynamics model was utilized to assess the gradation of influence on supply chain resilience and execute a single-factor sensitivity simulation analysis, further elucidating the dynamism and causative mechanisms of resilience within the PBSC. The outcomes of this research lay a foundation for devising management tactics that amplify supply chain resilience. Figure 1 delineates the methodological steps employed in this investigation.

SEM can simultaneously consider both direct and indirect relationships among multiple observed variables, thereby capturing complex networks of causality. For complex systems like the PBSC, SEM can provide insight into the interactions between influencing factors, as well as presenting the degree of significance of each factor in the form of quantitative data. In contrast, the SD model simulates and analyzes dynamic changes within a system and predicts future behavior. In PBSC systems, the SD method helps identify dynamic characteristics such as delay effects and feedback loops, and visualize the trend of toughness evolution in the form of images, thereby enhancing understanding of resilience evolution. Compared to using only SD-based simulation for resilience in PB projects or SEM methods for researching PBSC resilience factors and enhancement strategies^48,67^, the combined SEM-SD approach allows for an in-depth analysis based on existing theories and validates the model’s effectiveness and accuracy with real-world data. By integrating the results from these two approaches, combining qualitative and quantitative analysis, we can identify and quantify the key factors affecting supply chain resilience and simulate their performance under different scenarios, thereby providing more comprehensive decision support for supply chain management.

In developing approach, this study utilized SEM and SD models, drawing on theoretical underpinnings and reference values from previous studies. However, the integration of these two models to assess both static relationships and dynamic responses within PBSC represents a novel contribution of this work. This study extend the traditional use of SEM by incorporating data feeds into the SD model, enabling dynamic scenario analysis that more accurately reflects the complexity of the PBSC. Finally, this study propose further resilience enhancement strategies for PBSCs based on PSR theory, providing new insights for supply chain-related companies.

### Constructing an indicator system for resilience influencing factors in prefabricated building supply chain

Through academic search engines such as CNKI and Web of Science, using keywords such as "prefabricated building" and "supply chain" to search and collect high-quality literature related to the topic. In the process of literature analysis, factors such as technology, management and policy were paid attention to, and factors affecting the toughness of the PBSC were summarized, as shown in Table 2.

**Table 2:** Influencing factors of prefabricated building supply chain. Influencing factors of prefabricated building supply chain resilience are itemized in this table, categorized by their impact areas such as transportation, costs, and quality control. Accompanying each factor is a list of key references documenting their significance in the field. The table serves as a repository of research, aiding stakeholders in identifying the breadth of elements that can affect the supply chain, from economic shifts to raw material quality and legal policies.

Influencing factor	Theoretical explanation	References
Whether the transportation company has a credit	The reputation of transportation companies affects the timely and safe delivery of materials, ensuring the continuity and reliability of the supply chain	^3,70^
Design pattern change	Changes in design drawings can lead to adjustments in production and assembly processes, potentially causing delays and additional costs	^71–73^
Transportation cost	High transportation costs increase overall costs, impacting the economic efficiency and competitiveness of the supply chain	^6,73,74^
Rationality of the transportation standards	Reasonable transportation standards help improve efficiency and safety, reducing delays and damage	^6,45^
Duration control	Poor project progress control can lead to delivery delays, affecting the overall resilience of the supply chain	^75–77^
Safety misadventure	Safety incidents can cause production and transportation interruptions, leading to additional costs and delays	^77–79^
Warehousing expense	High storage fees increase overall costs, affecting the economic efficiency of the supply chain	^6,11,80^
Strategic planning accuracy	Precise strategic planning helps allocate resources effectively, reducing risks and uncertainties	^3,77^
Procurement price	Fluctuations in procurement prices can affect cost control and supply chain stability	^3,72,81,82^
Product quality	High-quality products reduce rework and repairs, improving the efficiency and reliability of the supply chain	^6,76^
Design scheme accuracy	Accurate design plans reduce modifications and errors during construction, facilitating smooth project execution	^77,83^
Information sharing	Effective information sharing enhances coordination and responsiveness within the supply chain, reducing delays and misunderstandings caused by information asymmetry	^11,45,72,84^
Project subcontracting	Poor subcontractor management can lead to quality issues and schedule delays, impacting the overall resilience of the supply chain	^41,77^
Supply chain structure	Complex supply chain structures increase the difficulty of coordination and the complexity of risk management	^11,41,45^
Manufacturing costs are too high	High manufacturing costs affect the economic efficiency of projects and the competitiveness of the supply chain	^77,85,86^
Changes in the economic environment	Changes in the economic environment can lead to demand fluctuations and supply chain disruptions	^11,76,87^
Raw material quality	Variability in raw material quality affects the final product quality and supply chain stability	^88,89^
Production Technology	Advanced production technologies improve efficiency and quality, enhancing supply chain resilience	^6,85,86,90^
Performance capacity of the supplier	The performance capabilities of suppliers directly affect the timeliness and quality of deliveries	^6,45,77,81^
Return maintenance	High costs of returns and maintenance increase the total cost of the supply chain, affecting economic efficiency	^6,73,85,86^
Quality of the completion acceptance is unqualified	Non-compliance with acceptance quality standards can lead to rework and delays, increasing project costs	^11,45,85,86^
Natural disasters	Natural disasters can disrupt the supply chain, complicating emergency response and recovery efforts	^11,41,76^
Market demand	Market demand fluctuations impact production planning and supply chain stability	^45,85,86^
Policy of the law	Changes in laws and policies can affect the compliance and operational efficiency of the supply chain	^73,83^

Based on these identified factors, a questionnaire was designed and disseminated. Respondents were asked to rate each factor on a scale of 1 to 5, where 1 indicated ‘irrelevant’, 2 ‘general’, 3 ‘somewhat important’, 4 ‘important’, and 5 ‘very important’. To ensure data reliability, respondents were selected from the construction-related industry. Using the ‘questionnaire star’ tool, 144 valid responses were collected. The majority of respondents were from collective and state-owned enterprises involved in PB. Notably, a significant proportion worked in general construction contracting and specialized construction contracting. Overall, the demographics suggest that the respondents’ feedback is reasonably informed. Detailed data can be found in Table 3.

**Table 3:** Information of respondents. Information of respondents from the survey encompasses various attributes such as enterprise type, involvement in prefabricated construction, years of employment, and business sectors. Percentages denote the distribution of respondents across categories like state-owned and foreign-funded enterprises, their experience span in the industry, and their roles ranging from general contracting to component parts production.

Project	Categories	Percentage
Type of working enterprise	Individual private enterprises	10.42%
	Collective enterprise	38.19%
	State-owned enterprises	34.03%
	Foreign-funded enterprise	17.36%
Whether the inaugural company participates in the prefabricated construction business	Yes	77.78%
	No	22.22%
Years of employment of the respondent	Within 1 year	17.36%
	1–3 Years	46.53%
	3–5 Years	23.61%
	More than 5 years	12.5%
Respondent business attributes	General contracting of construction construction	22.92%
	Professional contractors for construction	18.75%
	Development and construction	16.67%
	Survey and design	11.11%
	Component parts production	7.64%
	Other	22.92%

Data from the collected questionnaires were analyzed using SPSS to gauge respondents’ perceptions of factors influencing supply chain resilience in PB. As per Appendix 2, the mean scores for resilience factors ranged between 1.59 and 2.917, suggesting that most respondents deemed these factors as being between ‘general’ and ‘somewhat important’. The standard deviations, ranging from 0.865 to 1.226, highlight varied perceptions among respondents. Utilizing SPSS and the principal component analysis approach, factor analysis was conducted on 24 observed variables. Six primary components emerged, explaining 75.23% of the total variance—a value surpassing the threshold of 50%. This result underscores the efficacy of the adopted methodology. Appendix 2 provides more detailed data.

Applying the analytics-dimensional-reduction factor analysis function in SPSS 26.0, and after seven iterations, six common factors were identified. These factors represent a comprehensive understanding of the dataset. The distribution of these factors indicates their significant explanatory power concerning variance. The rotation matrix is documented in Table 4.

**Table 4:** Composition matrix after rotation. This table displays the rotated factor loadings for variables influencing the resilience of the prefabricated building supply chain. Values reflect the correlation strength of each factor with the components, with higher numbers indicating a stronger relationship. The matrix simplifies the interpretation by clustering related variables, thus helping to identify the most significant factors within each component.

The component matrix after the rotation						
	Ingredient					
	1	2	3	4	5	6
Whether the transportation company has a credit	0.824					
Design pattern change	0.774					
Transportation cost	0.774					
Rationality of the transportation standards	0.642					
Duration control	0.504					
Safety misadventure	< 0.5	< 0.5	< 0.5	< 0.5	< 0.5	< 0.5
Warehousing expense		0.818				
Strategic planning accuracy		0.814				
Procurement price		0.780				
Product quality		0.672				
Design scheme accuracy			0.832			
Information sharing			0.791			
Project subcontracting			0.740			
Supply chain structure			0.702			
Manufacturing costs are too high				0.838		
Changes in the economic environment				0.790		
Quality of raw materials does not meet the requirements				0.773		
Production technology does not meet the requirements				0.765		
Performance capacity of the supplier					0.882	
Return of goods maintenance ability is poor					0.852	
Quality of the completion acceptance is unqualified					0.804	
Natural disasters						0.823
Changes in market demand						0.767
Changes in laws and policies						0.764

From the analysis presented above, six distinct factors were identified using principal component analysis. Adhering to the criterion of eigenvalues exceeding 1, the cumulative contribution of these factors was 75.23%. Post-rotation, 23 observed variables exhibited values exceeding 0.5, which affirms the structural validity of the designed questionnaire. Excluding factors related to safety accidents, the 23 resilience-influencing factors were categorized into six primary indicators: A1 (transportation process factors), A2 (procurement process factors), A3 (planning factors), A4 (manufacturing process factors), A5 (delivery and utilization factors), and A6 (external environmental factors). This categorization facilitates the establishment of an index system for supply chain resilience influencing factors, detailed in Table 5.

**Table 5:** Index system of influencing factors of prefabricated building supply chain. This table categorizes the critical factors affecting the supply chain into a hierarchical index system. First-level indicators include component transport, procurement, planning, manufacturing, delivery and use, and external environmental factors. Each primary category is broken down into secondary indicators that specify the discrete elements like transportation company credit, cost of component transportation, and strategic planning accuracy, offering a detailed framework for analysis and improvement within the supply chain.

First-level indicators	Categories	Secondary indicators
A1 Component transport process factors	A11	Transportation company credit
	A12	Change of design drawings
	A13	Component transportation cost
	A14	Duration control
	A15	Reasonableness of transportation standards
A2 Procurement process factors	A21	Warehousing expense
	A22	Strategic planning accuracy
	A23	Procurement price
	A24	Product quality
A3 Planned factors	A31	Design scheme accuracy
	A32	Project subcontracting
	A33	Supply chain structure
	A34	Information sharing
A4 Manufacturing process factors	A41	Production cost is too high
	A42	Changes in the economic environment
	A43	Low quality of raw materials
	A44	Low production technology
A5 Delivery and use factors	A51	Performance capacity of the supplier
	A52	Poor return and maintenance ability
	A53	Unsatisfactory quality of acceptance
A6 External environmental factors	A61	Natural disasters
	A62	Changes in market demand
	A63	Changes in laws and policies

### Structural equation model

The structural equation models (SEM) excel in multivariate relationship analysis and are suitable for in-depth understanding of the complex relationships between different factors in the prefabricated construction supply chain. The method can be used to validate previously proposed theoretical models, ensuring that the underlying relationships and assumptions between variables in the model are supported. Using structural equation models, researchers were able to more fully analyze causality and gain insight into the impact of various factors in the PBSC on resilience.

SEM encompasses a set of mathematical equations, including structural equations that depict relationships between latent variables, as shown in Eq. (1), and measurement equations that connect latent variables with indicators, as demonstrated in Eqs. (2) and (3). This study’s data analysis comprises reliability and validity testing, and goodness-of-fit analysis.

1 $$ \eta = {\text{B}}\eta + \Gamma \xi + \zeta $$

2 $$ {\text{Y}} = \Lambda {\text{y}}\eta + \varepsilon $$

3 $$ {\text{X}} = \Lambda {\text{x}}\xi + \delta $$

In this study, the SEM model is constructed based on the in-depth analysis in “The theory of resilience in prefabricated building supply chains” of the impact of resilience at each stage of the PBSC. It systematically explores how resilience is influenced by different managerial and operational practices throughout the entire process, from raw material procurement to final product delivery. Additionally, the selection of secondary indicators is based on database literature and the theoretical support provided in Table 2. Table 2 details the influencing factors and their literature sources, ensuring a solid theoretical foundation for the selection of indicators. Using this methodological framework, a comprehensive SEM model was constructed to quantify and analyze the specific impact of different management practices on overall supply chain resilience in the context of PB. This rigorous, theory-based construction methodology enhances the transparency and scientific validity of the study.

Data collection was conducted through a questionnaire survey of practitioners in the PB industry, yielding 144 valid responses. The questionnaire employed a 5-point Likert scale to assess the importance of each factor. During the data analysis stage, descriptive statistical analysis and reliability tests were conducted using SPSS software, followed by SEM analysis using AMOS 23.0. The model’s reliability and validity were verified, and the model fit was assessed using Chi/df, GFI, AGFI, CFI, and RMSEA. Finally, the results of model fitting and path coefficients are analyzed. This methodology systematically identified the key factors affecting the resilience of the PBSC, providing a theoretical basis and empirical support for its management and optimization. Descriptive statistics were presented in “Constructing an Indicator system for resilience influencing factors in prefabricated building supply chain”, and subsequent sections will delve into reliability and validity tests.

#### Reliability test

The research designed a scale encompassing 23 items. To ensure scale consistency, the internal coherence of the scale was tested using 144 samples. This involved computing the Cronbach’s α value for the scale’s internal consistency reliability coefficient prior to exploratory factor analysis. Based on this, the measurement model’s reliability was ascertained through selection analysis, scale reliability analysis in SPSS. Generally, a Cronbach’s α value between 0 and 1 is acceptable, with values exceeding 0.8–0.9 signifying high reliability^91,92^.

As delineated in Table 6, the Cronbach’s α values for various factors such as construction and transportation processes, procurement, planning, manufacturing, delivery and utilization, and external environmental factors all surpass 0.75 and approach 0.9, indicating strong measurement reliability. With the coefficient set in SPSS as α > 0.6, the questionnaire’s α value is 0.937, and each latent variable’s α value exceeds 0.75. This underscores the validity and reliability of the collected data.

**Table 6:** The Cronbach’s α coefficient for the individual latent variable. The Cronbach’s α coefficient for the individual latent variable indicates the reliability of the constructs within the study, with values above 0.7 generally considered acceptable. This table shows the coefficient values for each of the supply chain process factors, reflecting the internal consistency of the responses.

Subactive variable	Observational variable	α coefficient
Component transportation process factors	5	0.875
Procurement process factors	4	0.898
Planned factors	4	0.873
Manufacturing process factors	4	0.882
Delivery and use factors	3	0.920
External environmental factors	3	0.796

#### Validity test

In SPSS, the Kaiser–Meyer–Olkin (KMO) value is utilized for validity testing, with a recommended threshold of KMO > 0.6^91,92^. For our data, the KMO was determined to be 0.886, affirming its suitability for factor analysis. Bartlett’s spherical test was employed to assess the independence of the questionnaire items, using a significance level of < 0.05 as the standard^91,92^. A derived significance value of 0.00 < 0.05 confirms that the questions in the questionnaire are adequately independent. Consequently, the data from the questionnaire survey adheres to the normal distribution and is apt for model fitting and path analyses. Detailed results are presented in Table 7.

**Table 7:** Results of the KMO test and the Bartlett spheroid test. Results of the KMO test and the Bartlett spheroid test provide a measure of the adequacy of the sample data for factor analysis. The KMO test results and the significant Bartlett’s test indicate that the questionnaire data is suitable for structure detection.

Metric	Result	
Questionnaire data	KMO checkout	
	KMO price	0.886
	Approximate chi square	2253.114
	Bartlett spherical test	
	df	253
	0.00	P-value

### System dynamics model

**Figure 2:**
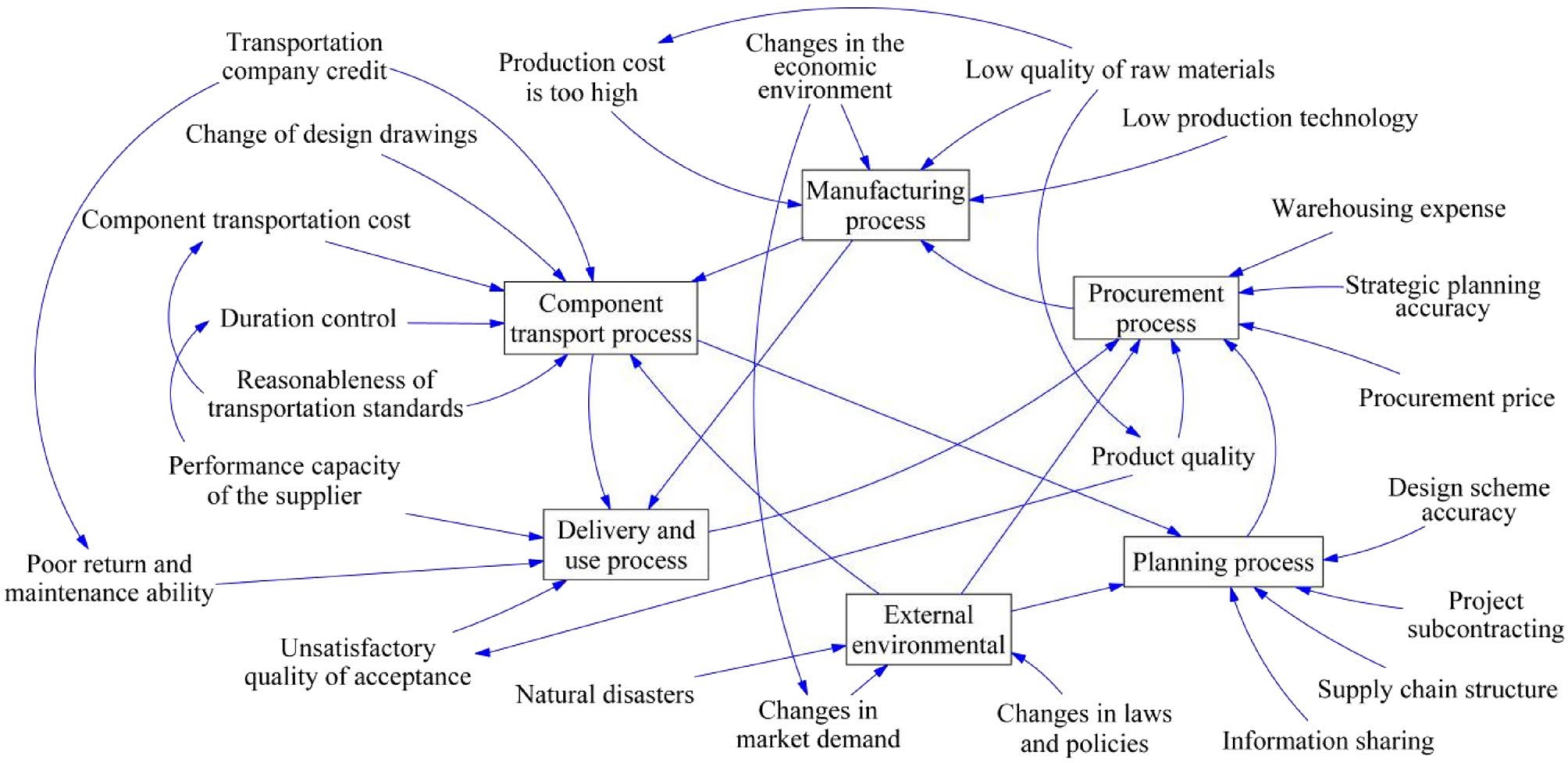
Causality diagram of influencing factors. This schematic illustrates the interplay between key elements within the supply chain for prefabricated buildings. It highlights how factors like production costs, material quality, and technological capability directly influence the manufacturing process, while external factors like economic conditions, market demand, and natural disasters shape the overall environment. The arrows depict the directional influence between components, revealing the interconnected nature of supply chain activities.

**Figure 3:**
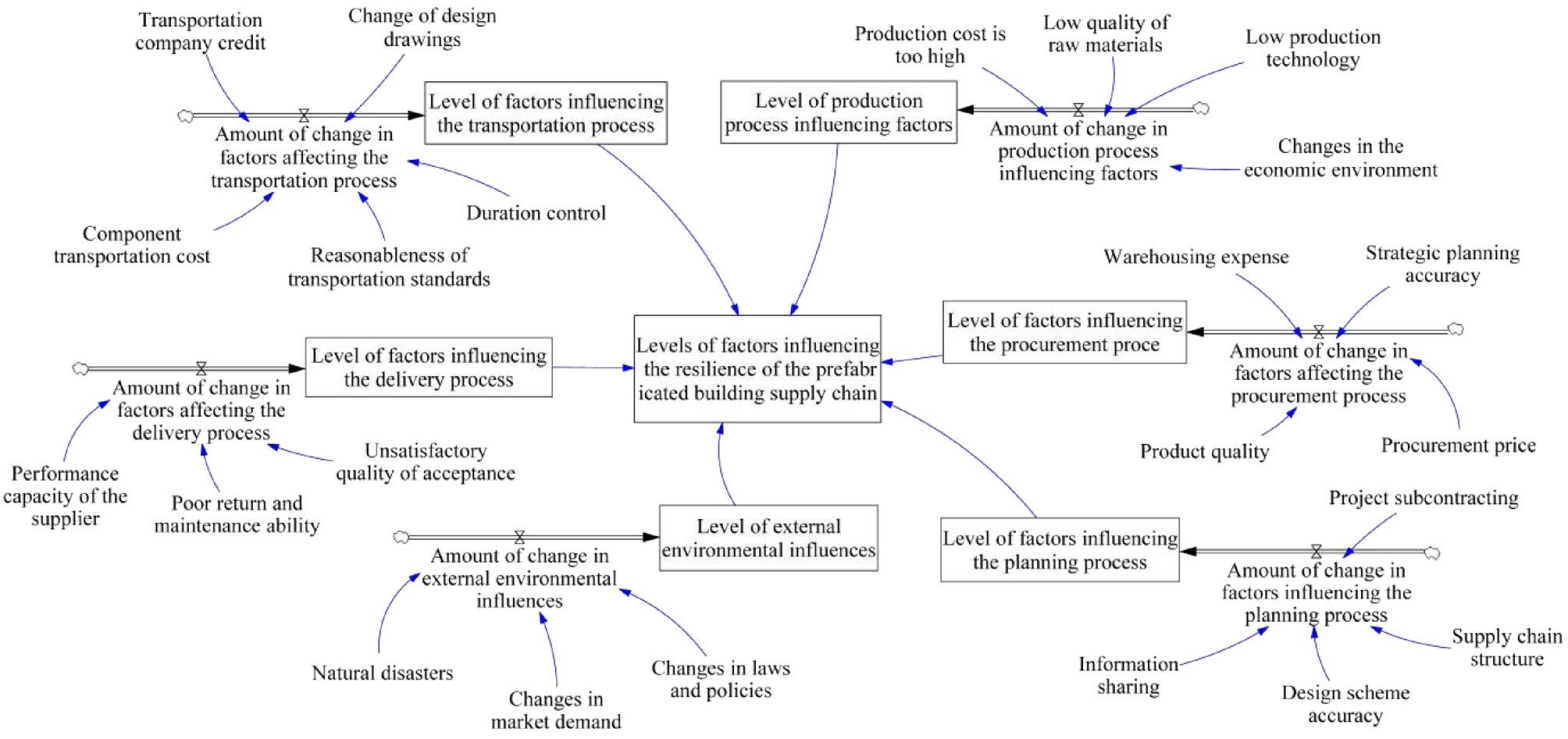
Impact factor stock flow diagram. This diagram provides a snapshot of the dynamic flows and levels within the prefabricated building supply chain. It shows the accumulation and depletion of various factors such as changes in laws and policies, information sharing, and supply chain structure, that collectively influence the resilience of the supply chain. The circles represent stocks of impact factors, and the arrows show the flow of changes or adjustments within the system.

The resilience of the supply chain for PB stems from the interplay of multiple factors. In examining these influencing elements, the System dynamics model delineates the boundaries of resilience factors, investigates their interrelations, and elucidates the nexus between risks and their interdependencies. Utilizing Vensim software, the causal loop diagram and stock flow chart for the resilience of PB supply were constructed, as illustrated in Figs. 2 and 3.

### PSR model

**Figure 4:**
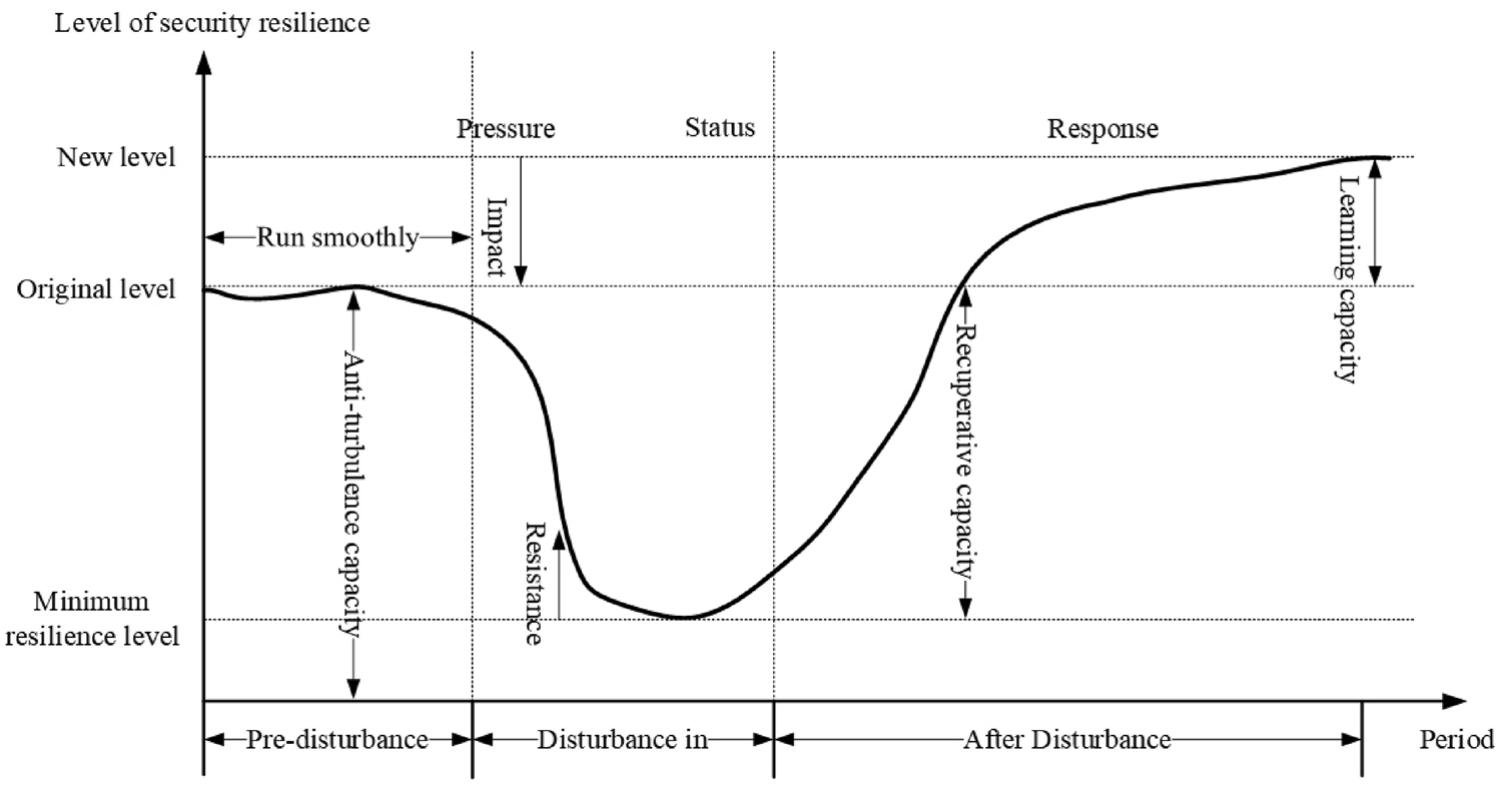
Perturbed PSR process for security resilience of prefabricated building supply chain. The graph depicts the resilience levels of a supply chain under the Pressure-State-Response framework. Initially, the system operates at its original level, signifying a smooth run. Upon encountering a disturbance, it experiences a drop to a minimum resilience level, indicating an absorption capacity challenge. In the status phase, resilience is assessed as the system’s ability to withstand pressure. As the system transitions to the response phase, it undergoes recovery and may reach a new level of resilience, ideally higher than the original due to adaptive measures and learned strategies. The x-axis represents the time period, illustrating the pre-disturbance stability, the impact of the disturbance, and the post-disturbance adaptation and learning processes.

The Pressure-State-Response (PSR) model offers an analytical framework tailored for environmental management and decision-making. Its design aims to holistically comprehend environmental challenges and their interplay with human endeavors^93^. In this research, "pressure" symbolizes external challenges faced by the PBSC; "state" represents the adaptive change in the chain’s safety resilience upon encountering stress; and "response" captures the subsequent reactions of the supply chain, as illustrated in Fig. 4. The PSR model captures the system’s dynamic interactions, resonating with the safety and resilience processes when the PBSC is perturbed. This enables enterprises in the PBSC to systematically discern potential risks, evaluate chain resilience, and derive strategies for enhancing safety resilience in a dynamic system state.

## Results and simulations

### Model fitting and mechanism analysis of resilience influential factors in prefabricated building supply chain

The study has successfully established a well-fitted model using SEM to explain the latent relationships among the influencing factors of PBSC research. Internal consistency tests indicate high levels of reliability and validity in the measurements, with analyses including construct reliability, factor analysis, and average variance extracted confirming the rationality of the measurement model. This is indicative of the model’s scientific validity and rationality. Three comprehensive indices—absolute fit index, relative fit index, and parsimony fit index—are employed to assess the model’s fit. Detailed metrics are presented in Table 8.

**Table 8:** Fitting results of the structural equation models. Fitting results of the structural equation models are summarized here, showcasing the model’s goodness-of-fit to the observed data. Acceptable fit indices suggest the model’s estimates are consistent with the expectations set by the theoretical framework.

Fitting the index	Metric	Reference to standard^91,92^	Fits the result	Matching result
Absolute fit index	Chi/df	1–3	1.752	Accept
	GFI	> 0.8	0.813	Accept
	RMSEA	< 0.08	0.073	Accept
Relative the fit index	IFI	> 0.9	> 0.9	Accept
	TLI	> 0.9	0.912	Accept
	CFI	> 0.9	> 0.9	Accept
Streamlining the fit index	PCFI	> 0.5	0.816	Accept
	PNFI	> 0.5	0.741	Accept

**Figure 5:**
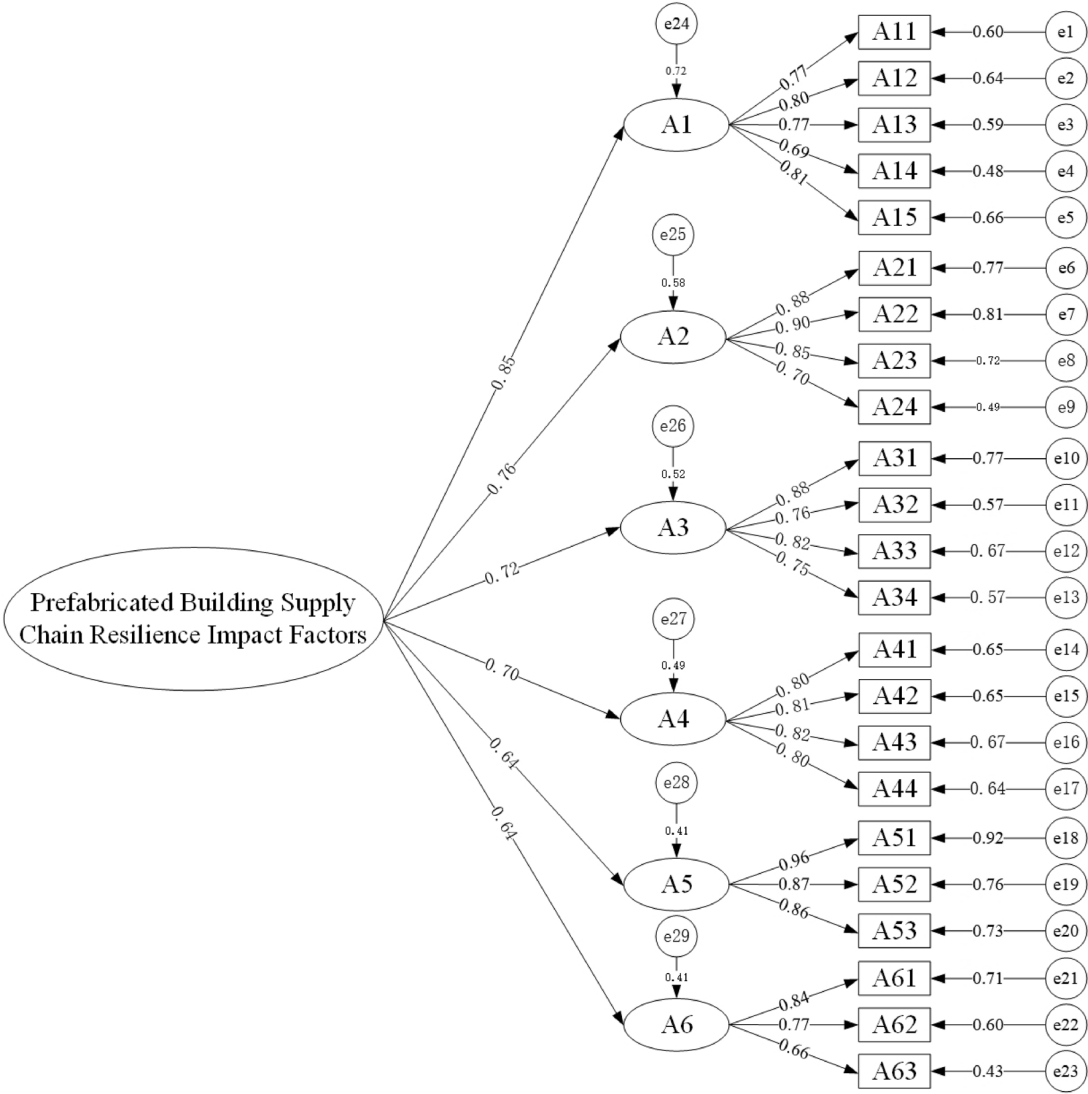
Structural equation modeling of impact factors. This diagram visualizes the complex relationships and relative strengths among various impact factors affecting the resilience of the prefabricated building supply chain. Each circle, labeled A1 through A6, represents a latent variable or group of factors, with the corresponding rectangles (A11 through A63) depicting observed variables. The directed arrows from latent to observed variables signify hypothesized influences, with the values beside the arrows indicating the strength and direction of these relationships. The higher the absolute value, the stronger the influence. Error terms (e1 through e23) associated with observed variables account for measurement error or unexplained variance. This model provides a quantitative representation of how multiple factors interconnect to determine the overall resilience of the supply chain.

Post-evaluation, all fit indices aligned with the established standards, and the fit outcomes were deemed acceptable. The AMOS software was harnessed to construct the structural equation model for supply chain resilience. Within this model, post-adjustment, six latent variables alongside their path coefficients were derived, facilitating the computation of each latent variable’s weight ratio. This analysis aids in pinpointing pivotal factors that shape supply chain resilience, furnishing invaluable insights for decision-makers, as illustrated in Fig. 5 and Table 9.

**Table 9:** The weight ratios of the first-level indicators and second-level indicators calculated by the normalization method. The weight ratios of the first-level indicators and second-level indicators calculated by the normalization method reflect the relative importance of various factors influencing the resilience of the prefabricated building supply chain. Each primary indicator, such as component transport process factors and procurement process factors, is assessed along with more detailed secondary indicators like transportation company credit and strategic planning accuracy, providing a quantified hierarchy of impact within the supply chain structure.

Name	First-level indicators	Weight ratio	Secondary indicators	Weight ratio
Prefabricated building supply chain resilience influencing factors	A1 Component transport process factors	0.197	A11 Transportation company credit	0.201
			A12 Change of design drawings	0.208
			A13 Component transportation cost	0.201
			A14 Duration control	0.179
			A15 Reasonableness of transportation standards	0.211
	A2 Procurement process factors	0.176	A21 Warehousing expense	0.264
			A22 Strategic planning accuracy	0.270
			A23 Procurement price	0.255
			A24 Product quality	0.211
	A3 Planned factors	0.167	A31 Design scheme accuracy	0.275
			A32 Project subcontracting	0.257
			A33 Supply chain structure	0.234
			A34 Information sharing	0.234
	A4 Manufacturing process factors	0.162	A41 Production cost is too high	0.245
			A42 Changes in the economic environment	0.249
			A43 Low quality of raw materials	0.252
			A44 Low production technology	0.245
	A5 Delivery and use factors	0.149	A51 Performance capacity of the supplier	0.357
			A52 Poor return and maintenance ability	0.320
			A53 Unsatisfactory quality of acceptance	0.323
	A6 External environmental factors	0.149	A61 Natural disasters	0.342
			A62 Changes in market demand	0.289
			A63 Changes in laws and policies	0.369

Data analysis from the aforementioned table reveals that among the influencing factors of the PBSC, component transportation factors exert the most significant influence at 0.197. This is closely followed by procurement process factors at 0.176. This is followed by planning (0.167), manufacturing process (0.162), delivery and use (0.149), and the influence of external environmental factors (0.149). Subsequent system dynamics simulations will be based on these findings.

### Evaluation of resilience impact levels and sensitivity analysis

#### Initial setting of the system dynamics model

To analyze the factors impacting the resilience of the PBSC, a simulation project with a duration of eight weeks and a 1-week step interval was established. The constant estimation data for this model were derived from SEM, mean value analysis, and logical estimations to ascertain corresponding risk estimates. Additionally, the normalized weight coefficients of the primary indicators in the SEM were transformed into values for the corresponding variables in the SD model. Appendix 3 enumerates the estimated values and equations for the 23 system boundary constants.

#### Model test

Subsequently, the model underwent a series of evaluations, including system boundary testing, consistency testing, and extreme condition testing. System boundary testing ensures the effectiveness of the model within the system scope, validating its comprehensive capture of system dynamics. Consistency testing evaluates the logical consistency among various internal components of the model, ensuring its overall structure is rational. Extreme condition testing examines the model’s performance under extreme circumstances, verifying its robustness and reliability across various scenarios. Through these tests, we have confirmed the effectiveness of the SD model. The model was evaluated for its validity under extreme conditions, using variables related to the transportation process level as an example. The standard values for these variables lie within the range of 0 and 1. Different values (0, 0.15, 0.5, and 1) for the variables related to the transportation process level were selected to observe the changes in the resilience of the supply chain for PB. The simulation results indicated that when the "transportation process" is set to 1, the resilience of the supply chain reaches its peak, while it is at its minimum when the "transportation process" is set to 0. The resilience is positively correlated with the transportation process, and the simulation results are consistent with the actual conditions. Additionally, other variables of the model also successfully underwent verification. The results from these tests confirmed that all requirements were satisfactorily met.

#### Evaluation of resilience impact levels

**Figure 6:**
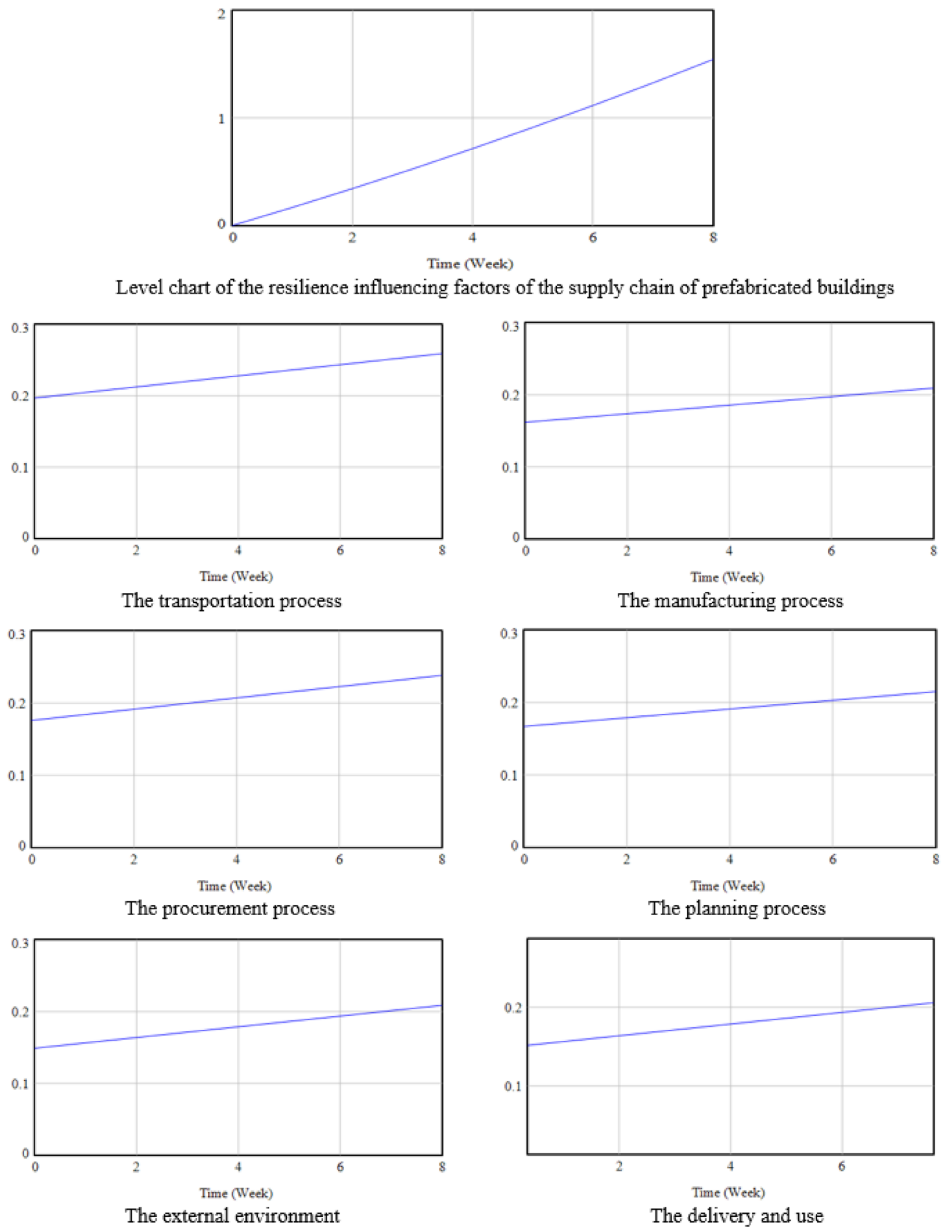
Trend chart of the dynamics of the level of factors affecting the resilience of the prefabricated building supply chain. This set of trend charts depicts varying levels of resilience across different stages of the supply chain for prefabricated buildings over an eight-week period. The overarching trend, represented in the top chart, indicates an overarching increase in resilience levels, with fluctuations that suggest varying degrees of impact across different phases. Individual charts for the transportation, manufacturing, procurement, planning, external environment, and delivery and use processes show differing trends. While some processes exhibit a steady increase in resilience, indicating a robust response to disturbances, others maintain a flatter line, implying a more moderate enhancement of resilience. These variations highlight the disparate levels of sensitivity and capacity for recovery inherent to each segment of the supply chain. The x-axis uniformly measures time across all charts, and the y-axis represents the relative level of resilience, showcasing the nuanced impact each factor has on the system’s overall robustness.

Analysis of resilience influencing factors in PB aims to forecast the likelihood and magnitude of such factors, identify primary sources, and subsequently select preventative or control measures. This facilitates the maximal enhancement of supply chain resilience. Vensim software was employed for model simulation, yielding the dynamic trend diagram of factors influencing the resilience of the PBSC, as illustrated in Fig. 6. Each starting point on the graph represents the initial impact of resilience factors at the beginning stage of the engineering project, while the slope indicates the growth rate of each factor over time.

Examination of the resilience impact trends reveals that as the construction project advances, the effects of the resilience influencing factors on the PBSC intensify, peaking upon project completion. This suggests the inevitability of the event. A closer examination of the trend graphs for the PBSC resilience subsystems reveals that, among the six subsystems, the procurement and transportation processes exhibit the highest rates of change over time, while the other processes show lower rates of change. This dynamic perspective helps identify critical processes and potential risk points to focus on in actual operations.

#### Sensitivity analysis of resilience influencing factors

**Figure 7:**
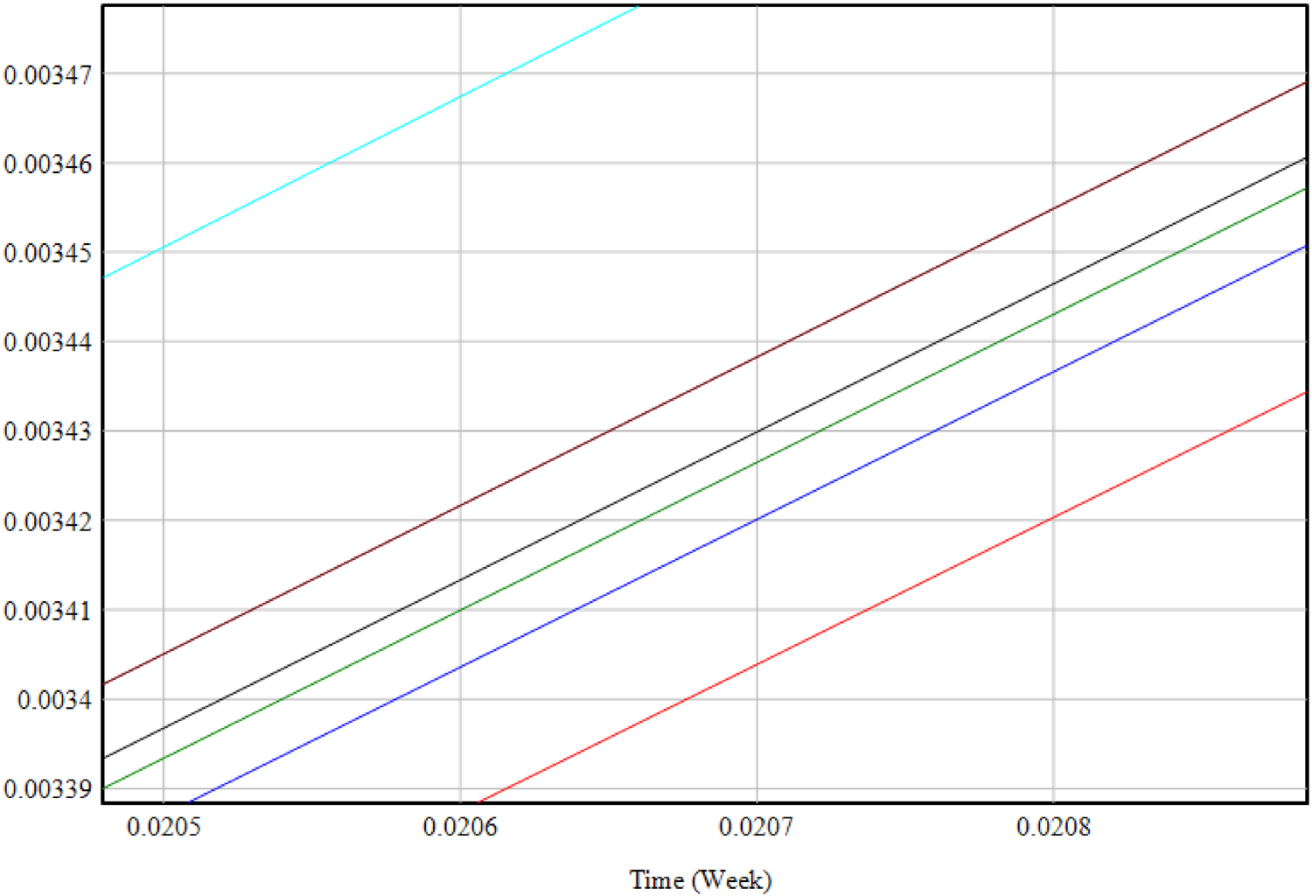
Trend chart of the level of factors influencing the resilience of prefabricated building supply chain after single factor change. The graph illustrates the trends in resilience levels in response to changes in individual factors over time, represented in weeks. Each line corresponds to a specific factor within the supply chain. The various colors differentiate between the factors, with each line tracing the progression of resilience levels as they are impacted by a single factor change. The temporal axis, scaled in weeks, captures a snapshot of the resilience trajectory, underscoring the varying degrees of sensitivity to changes across different supply chain components.

To discern the sensitive factors affecting the resilience of the PBSC, certain factors were held constant while others, namely the transportation, procurement, manufacturing, planning, delivery and use processes, and external environmental factors, underwent a decremental simulation by 10%. A solitary factor was adjusted in each subsystem, and the subsequent levels of resilience under these perturbations across 7 states are depicted in Fig. 7.

Observing Fig. 7, the hierarchical sequence from top to bottom represents the current state, followed by external environmental influence, delivery and use process, manufacturing process, planning process influence, procurement process influence, and transportation process influence. When each factor’s weight is reduced by 10%, both the transportation and procurement processes exhibit the most pronounced deviations. The transportation process displays the largest divergence from the baseline, highlighting its paramount importance in the resilience analysis of the PBSC. Subsequent processes in descending order of significance include procurement, planning, manufacturing, delivery and use, and external environmental influences. The trajectories for the manufacturing process and the delivery and use process closely align, necessitating a more granular analysis in Vensim’s Time Table. Table 10 enumerates the value shifts resulting from single factor modifications.

**Table 10:** Data sheet on changes in factors affecting resilience. This table records the quantitative impact of different factors on the resilience of the prefabricated building supply chain over an eight-period timeframe. It traces the progression of effects from transportation, manufacturing, procurement, external environment, planning, and delivery and use, with initial values at time zero and subsequent measurements at regular intervals, highlighting the evolving nature of supply chain resilience.

Time	Current	Effect of transportation process	Manufacturing process influence	Effect of procurement process	External environmental influence	Planning process influence	Delivery and use process
0	0	0	0	0	0	0	0
1	0.168320	0.164439	0.165696	0.165222	0.166100	0.165531	0.166100
2	0.343733	0.335818	0.338388	0.337401	0.339182	0.338055	0.339182
3	0.526240	0.514137	0.518078	0.516534	0.519245	0.517572	0.519248
4	0.715839	0.699395	0.704765	0.702624	0.706291	0.704081	0.706296
5	0.912532	0.891594	0.898449	0.895669	0.900318	0.897584	0.900326
6	1.116320	1.090730	1.099130	1.095670	1.101330	1.098080	1.101340
7	1.327200	1.296810	1.306810	1.302630	1.309320	1.305570	1.309340
8	1.545170	1.509830	1.521480	1.516540	1.524290	1.520050	1.524310

The data table suggests minimal alterations in the external environmental influences and the delivery and use process relative to the baseline. However, a deeper examination reveals a diminished sensitivity of the external environmental factors in comparison to those of the delivery and use process. Consequently, the ranked sensitivity of the factors from highest to lowest is as follows: transportation process, procurement process, planning process, manufacturing process, delivery and use process, and external environmental influence.

### PSR model-based security resilience enhancement strategy for prefabricated building supply chain

**Figure 8:**
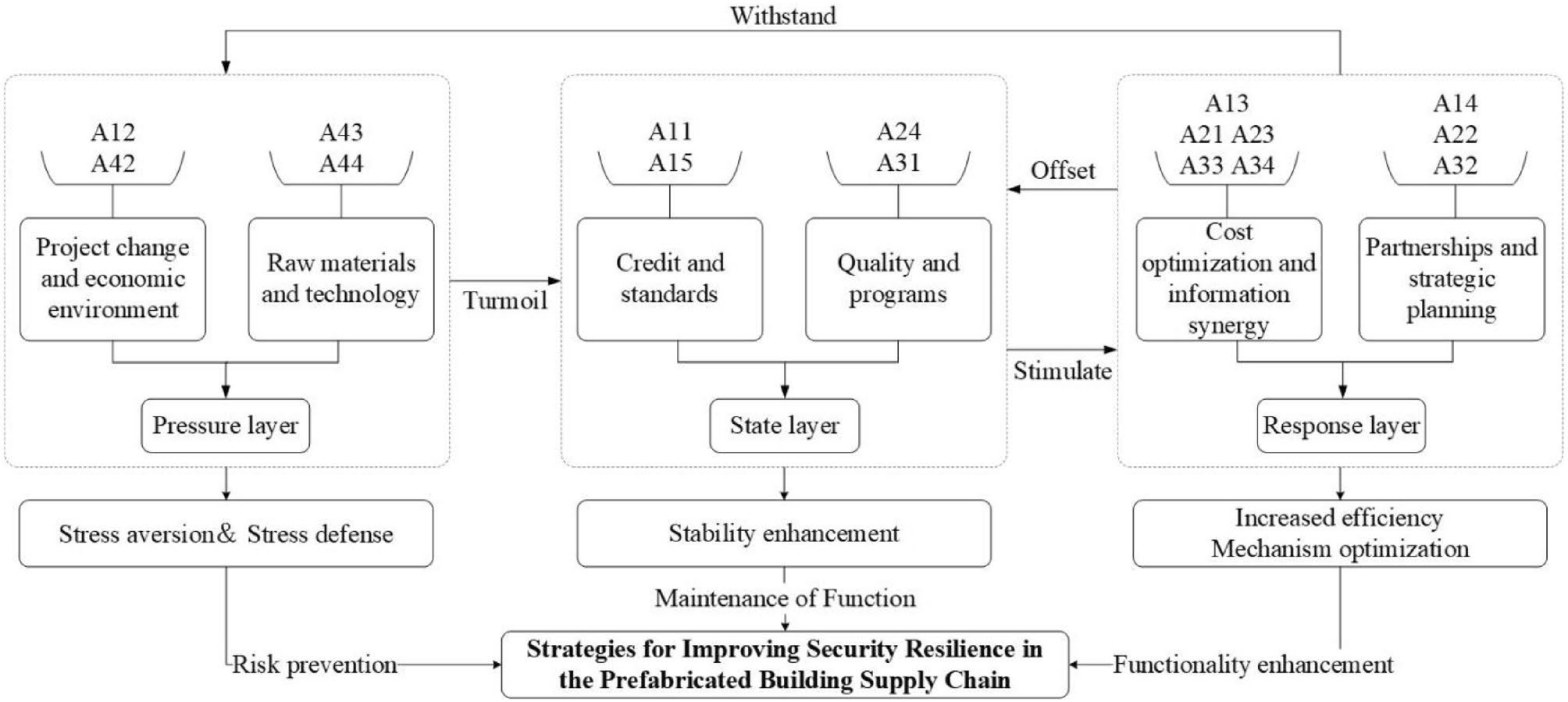
A Strategic framework for security resilience improvement in the prefabricated building supply chain. This diagram outlines a three-tiered approach to bolstering resilience, categorized into the Pressure, State, and Response layers. The Pressure layer, driven by project changes and the economic environment (A12, A42) as well as raw materials and technology (A43, A44), is where initial stressors are identified. The State layer, depicted in the center, addresses the system’s current condition, focusing on maintaining credit and standards (A11, A15) and quality and programs (A24, A31). The Response layer encompasses strategies like cost optimization and information synergy (A13, A21, A23, A33, A34) and partnerships and strategic planning (A14, A22, A32), aimed at enhancing the system’s functionality and efficiency. The connecting arrows suggest a flow from recognizing pressures, stabilizing the current state, to implementing responsive strategies, all contributing to a comprehensive resilience improvement strategy.

Gleaning insights from the structural equation model and system dynamics outcomes, the transportation, procurement, planning, and manufacturing processes emerge as the pivotal influencing processes of the PBSC. In alignment with the pressure-state-response (PSR) model delineated in “PSR Model”, the 16 resilience indicators from these four processes are categorized into the pressure, state, and response layers of the PSR model. Specifically, the pressure and state layers comprise 4 indicators each, whereas the response layer encompasses 8 indicators. This classification aids in constructing the safety and resilience improvement strategy framework, as illustrated in Fig. 8.

#### Explanation of security resilience improvement strategies for prefabricated building supply chains

Implementing pressure layer defense is an effective method for enhancing the security resilience of supply chains. This defense strategy encompasses aspects: Project change and economic environment, Raw materials and technology. (1) Project change and economic environment: establish a stricter review and change process. Establish a specialized team to focus on government policies and track economic trends. Proactive defense measures, For instance, Bhattacharya et al.^94^ concluded that establishing an emergency fiscal reserve can help alleviate potential economic disruptions. (2) Raw Materials and Technology: Adopting the strategy of "one to many" and establishing long-term cooperative relationships with several suppliers. Develop new technologies and processes to improve production efficiency.

State layer recovery serves as a crucial method for upholding the resilience of supply chain security. This recovery is categorized into two main areas: Credit and standards, Quality and programs. (1) Credit and standards: Conducting credit background checks on partners, utilizing a credit rating model, and regularly evaluating their credit rating from a financial perspective^95^. Comprehensive and pragmatic transportation guidelines should be instituted, with periodic revisions to reflect evolving market dynamics and technological advancements. (2) Quality and programs: Efforts can be directed towards establishing an integrated synergy between quality management and supply chain management, encompassing both performance enhancement and integrated improvement methodologies^96^. Iterative deliberations on design methodologies and ongoing team training ensure alignment with the industry’s apex standards.

Enhancing the response layer is an effective strategy for increasing supply chain security resilience. This enhancement focuses on two key areas: Cost optimization and information synergy, Partnerships and strategic planning. (1) Cost optimization and information synergy: Conduct cost analysis and optimize purchasing and storage processes. Implement lean production, optimize the supply chain structure. The company can integrate best practices of both financial and non-financial collaborative initiatives into the synergistic functioning of its supply chain, spanning from production design, procurement, and inbound logistics to manufacturing processes, distribution, and outbound logistics^97^. (2) Partnerships and strategic planning: Faced with growing risks, businesses are increasingly inclined to consciously form partnerships and engage in supply chain collaboration, where the level of cooperation among partners contributes to the resilience of the supply chain^98^. Select experienced subcontractors and establish strict contract terms and assessment standards. Instituting a cross-functional strategic planning committee ensures holistic insights and feedback. Regularly evaluate and revise strategic plans to respond to market and internal changes.

## Discussion

This study advances the field of PBSC resilience by introducing an integrated SEM and SD approach, which has not been thoroughly explored in the existing literature. Unlike previous models that focused solely on static analysis, our approach captures both static and dynamic elements of supply chain resilience, resulting in a more comprehensive understanding of how resilience factors evolve over time. Traditional SEM methods, as used in studies Zhang et al.^6^ and Qi et al.^66^, focus on static relationships between variables. While these approaches are robust in identifying key influencing factors, they fall short in explaining dynamic interactions over time. In contrast, standalone SD models, as employed in studies Zhang et al.^45^ and MacAskill et al.^99^, effectively model dynamic systems but typically lack explicit quantification of causal relationships between underlying variables. Our integrated SEM-SD approach outperforms traditional SEM and standalone SD models by not only quantifying the significance of resilience-influencing factors but also revealing the degree of resilience change in each factor over time. This superior performance highlights the effectiveness of integrating static and dynamic analyses for a comprehensive understanding of PBSC resilience, thereby contributing to both theoretical and practical capabilities in managing PBSC.

Based on structural equation modeling, it helps to achieve a comprehensive understanding of the key factors of supply chain resilience. Through a comprehensive analysis using structural equation modeling, combined with single-factor sensitivity analysis, it is evident that the transportation process exhibits the most significant deviation from the initial curve. Moreover, it stands out as the most sensitive factor. This highlights the transportation process’s influential role as a critical determinant in assessing the resilience of the PBSC. However, the study by Zhang et al.^6^ suggested that the transportation and storage processes of the components did not significantly affect supply chain resilience. This contrasts with the results of this study and indicates that future theoretical research should further explore the effects of the transportation process under various environments and conditions. Despite Zhang et al.’s finding of no significant impact, this study reveals the critical role of the transportation process in the PBSC through structural equation modeling and one-factor sensitivity analysis. According to Meyer et al.^100^, transportation plays a pivotal role in the response to supply chain disruptions, particularly within the prefabricated construction supply chain. In this context, transportation is not only critical for the timeliness and cost control of materials but also directly linked to the coherence and reliability of the supply chain. This perspective further underscores the significance of the transportation process as a key factor in the resilience of the prefabricated construction supply chain^100^.

Ranking the determinants affecting supply chain resilience, from the most influential to the least, they are as follows: transportation process (0.197), procurement process (0.176), planning process (0.167), manufacturing process (0.162), delivery and usage (0.149), and external environmental influences (0.149). These influencing factors are notably concentrated, particularly in procurement, planning, and manufacturing processes, which are more significant than other factors. According to a previous study by Cai et al.^68^, the procurement process is also an important factor influencing the risk of the PBSC. The study by Chen et al.^101^ reveals that management decisions in procurement and manufacturing processes, especially regarding alternative sourcing, are crucial for mitigating the financial risks caused by supply and demand uncertainties. This finding provides further theoretical support for the idea that procurement is not only central to supply chain operations but also a key factor in shaping supply chain resilience. The findings derived from the above SEM help to identify and recognize the key factors affecting supply chain resilience and provide a static, quantitative basis for the development of management strategies.

The SD model offers a dynamic analysis method by simulating the recovery behavior of the supply chain in response to external perturbations. Compared to the findings from the SEM, this approach illustrates the resilience changes of the supply chain over time, effectively capturing its ability to cope with shocks at different points in the timeline, and providing guidance for supply chain design and emergency preparedness^67^. In assessing the resilience impact level of the PBSC, resilience impact factor events are likely to emerge as the project progresses, with the procurement and transportation phases exhibiting significant resilience changes over an 8-week period. Interestingly, our findings reveal that external environmental factors exert a more pronounced influence compared to the delivery and use process factors. However, when focusing on the one-factor sensitivity analysis, the delivery and use process factors appear to be more sensitive than their external environmental counterparts.

Consequently, external environmental factors play a more pronounced role in the overall assessment of resilience in the prefabricated construction supply chain. This significance further supports a positive moderating relationship between supply chain agility and supply chain adaptability^102^. For instance, adverse weather conditions can precipitate delays in the timely arrival of prefabricated components, directly impinging on the entire construction workflow. Conversely, the delivery process emerges as more significant in the sensitivity analysis, this is primarily due to intrinsic factors such as technical nuances in logistics support levels and operational subtleties in collaboration intensity^67^. These can occasionally culminate in pronounced setbacks and unforeseen expenses; a case in point being an error in installing a crucial component that necessitates remanufacturing. This result has significant theoretical implications as it challenges the widespread emphasis on external environmental factors in traditional theories^11^. It emphasizes the critical impact of internal process management, such as delivery and usage processes, on supply chain resilience in practice.

In the quest to bolster the security resilience of the PBSC, the PSR theory has been adeptly applied. This innovative approach identifies transportation, procurement, planning, and manufacturing processes as pivotal impact factors within the PBSC. Within these four processes, the 16 resilience indicators are systematically stratified into pressure, state, and response layers. Subsequently, an array of targeted and actionable enhancement strategies and measures has been delineated. Beyond their academic implications, these strategies present substantial pragmatic relevance. It is anticipated that they will serve as valuable resources for both scholars and practitioners in the PB domain.

## Conclusion

This research endeavors to undertake a thorough analysis of the factors influencing supply chain resilience within China’s PB sector. The objective is to enhance the adaptability and risk mitigation capacities of operations within pertinent supply chain entities. The research begins by conducting a comprehensive literature review and analysis, complemented by the utilization of SPSS statistical software and Structural Equation Modeling (SEM). Through statistical validation, a robust resilience indicator system for the PBSC is constructed, comprising 6 primary indicators and 23 secondary indicators. Subsequently, the SEM is employed to delve into the intricate relationships among various factors influencing the resilience of the PBSC. This in-depth exploration facilitates a comprehensive understanding of the key factors affecting supply chain resilience, shedding light on the internal mechanisms employed when facing external shocks and changes.

The research identifies a concentration of importance in certain factors, with transportation process impact and procurement process impact highlighted as particularly significant, constituting weight proportions of 0.197 and 0.176, respectively. The resilience impact factors are then ranked in descending order from planning process impact to manufacturing process impact, delivery and utilization impact, and external environmental impact. Following this, a System dynamics model is established to predict the temporal trends of resilience impact factors. The study reveals that as the project progresses, the effects of resilience impact factors in the PBSC steadily increase. The most pronounced rates of dynamic change are in the procurement process and transportation process factors, with the rest of the processes having lower rates of change. The predictive analysis of the temporal evolution of resilience factors enables the early detection of potential risks and challenges. This allows managers to proactively implement preventive measures, enhancing the supply chain’s adaptability. Understanding the temporal trends of resilience factors also aids in formulating longer-term strategic plans, adjusting and optimizing the supply chain’s long-term strategies to better adapt to future market and environmental changes. Simulating the changing trends of resilience factors enables more effective optimization of resource allocation and management, improving overall supply chain resilience.

Lastly, a sensitivity analysis is conducted on the resilience impact factors in the PBSC by systematically reducing each subsystem’s various factor values by 10%. The results indicate that changes in the transportation process impact have the most significant influence on the entire system’s resilience impact factors, followed by procurement process impact, planning process impact, manufacturing process impact, delivery and utilization process impact, and external environmental impact. Remarkably, the external environmental factors wielded a more pronounced impact on the overall evaluation of supply chain resilience than the delivery and use processes. However, the sensitivity of the latter surpassed that of the external environmental influences. The single-factor sensitivity analysis helps determine the impact of each parameter on the system output, identifying key parameters that guide targeted system management. In practical applications, it provides guidance for resource allocation and decision optimization, enabling decision-makers to make more informed resource management decisions. Additionally, the study proposes safety resilience enhancement strategies based on the Pressure-State-Response (PSR) model, offering valuable insights for practitioners in the PB industry to understand and address supply chain resilience impact factors.

This study provides a comprehensive analysis of the factors affecting PBSC resilience by integrating SEM and SD. This integrated approach offers important theoretical advancements and practical contributions, addressing the limitations of traditional methods through the dynamic and static combination, and establishes a robust framework for future research. The findings challenge the prevailing emphasis on external environmental factors in traditional theories and highlight the critical impact of internal process management on supply chain resilience in practice. The theoretical view that transportation and procurement stages are key resilience factors is further supported, emphasizing their importance in maintaining supply chain stability and continuity. Dynamic simulation and sensitivity analysis enable stakeholders to anticipate potential disruptions and proactively mitigate risks. The PSR based model provides actionable insights, emphasizing the importance of targeted interventions in transportation and procurement to enhance resilience. Overall, the study presents theoretical advancements and practical solutions that can help improve the resilience and sustainability of supply chains in the PB industry.

Although the objectives of this study were achieved, some limitations were still present. As the field of PB evolves, there remains a continual need to refine the factors influencing supply chain resilience. Larger datasets are essential to improve the accuracy of the model. Future research directions should address these limitations by delving deeper into less-explored factors within the PBSC, such as cultural differences and policy regulations, for a more comprehensive understanding of their resilience impact. In addition, by applying the model developed in this study to a real case study, more specific supply chain resilience can be guided. In short, the insights gleaned from this investigation hold significant value. They equip relevant enterprises with tools to more adeptly identify, evaluate, and address resilience challenges within the PBSC, ultimately refining operational models and fostering superior supply chain systems.

### Supplementary Information

Supplementary Information.

## Data Availability

The data underlying the results presented in the study are included within the manuscript.
