# Supplementary material for: Analyzing resilience influencing factors in the prefabricated building supply chain based on SEM-SD methodology
Source: Sci Rep. 2024 Jul 29;14:17393. doi: 10.1038/s41598-024-65271-2 (PMC11286961; doi:10.1038/s41598-024-65271-2)
Supplement: Supplementary file 1 — Supplementary Information. [file 41598_2024_65271_MOESM1_ESM.docx]

**Appendix A**

**Analyzing Resilience Influencing Factors in the Prefabricated Building Supply Chain Based on SEM-SD Methodology**

Dear Mr. / Madam,

Thank you for taking the time to participate in this survey. This inquiry forms part of an academic research titled "Analysis of Influence Factors of Prefabricated Building Supply Chain." Please be assured that the information you provide will be utilized strictly for academic purposes, and neither personal nor business details will be disclosed. We greatly appreciate your contribution and any additional insights you may provide. Once again, thank you for your invaluable support.

1. What kind of organization do you work for now?[single choice]*

| ○ State-owned enterprises |
| --- |
| ○ Collective enterprise |
| ○ Foreign-funded enterprise |
| ○ Individual and private enterprises |

2. What is your gender?[single choice]*

| ○ Man |
| --- |
| ○ Woman |

3. Does your company use prefabricated buildings?[single choice]*

| ○ Yes |
| --- |
| ○ No |

4. How long have you been engaged in the industry?[single choice]*

| ○ Within 1 year |
| --- |
| ○ 1-3 years |
| ○ 3-5 years |
| ○ For 5 + years |

5. Business attributes [Multiple choice] *

| ○ Development and construction |
| --- |
| ○ Exploration design |
| ○ General contracting of the construction works |
| ○ Professional contractors for the construction projects |
| ○ Supervision |
| ○ Materials, equipment, parts supply |
| ○ Components production |
| ○ Engineering Consulting |
| ○ Scientific research institution |

2. This questionnaire is aimed at the evaluation stage of the influence factors of the supply chain of prefabricated buildings,

Please evaluate the risk level of the following secondary risk indicators. As shown in Table 1, select whether the risk indicators meet the different risk levels. If so, type "√" in the table.

Table A.1 Questionnaire

|  | Have nothing to do with | Same as | More important | Important | Very important |
| --- | --- | --- | --- | --- | --- |
| Changes in laws and policies | ○ | ○ | ○ | ○ | ○ |
| Natural disasters | ○ | ○ | ○ | ○ | ○ |
| Changes in market demand | ○ | ○ | ○ | ○ | ○ |
| Changes in the economic environment | ○ | ○ | ○ | ○ | ○ |
| Manufacturing costs are too high | ○ | ○ | ○ | ○ | ○ |
| Production technology is not enough to meet the quality requirements | ○ | ○ | ○ | ○ | ○ |
| Low quality of building raw materials does not meet the requirements | ○ | ○ | ○ | ○ | ○ |
| Design pattern change | ○ | ○ | ○ | ○ | ○ |
| Whether the transportation company has a credit | ○ | ○ | ○ | ○ | ○ |
| Transportation cost of assembly parts | ○ | ○ | ○ | ○ | ○ |
| Rationality of the transportation standards | ○ | ○ | ○ | ○ | ○ |
| Project subcontracting | ○ | ○ | ○ | ○ | ○ |
| Information sharing | ○ | ○ | ○ | ○ | ○ |
| Design scheme accuracy | ○ | ○ | ○ | ○ | ○ |
| Supply chain structure | ○ | ○ | ○ | ○ | ○ |
| Strategic planning accuracy | ○ | ○ | ○ | ○ | ○ |
| Product quality | ○ | ○ | ○ | ○ | ○ |
| Warehousing expense | ○ | ○ | ○ | ○ | ○ |
| Performance capacity of the supplier | ○ | ○ | ○ | ○ | ○ |
| Poor return and maintenance ability | ○ | ○ | ○ | ○ | ○ |
| Quality of the completion acceptance is unqualified | ○ | ○ | ○ | ○ | ○ |
| Safety misadventure | ○ | ○ | ○ | ○ | ○ |
| Duration control | ○ | ○ | ○ | ○ | ○ |

**Appendix B**

Table B.1 Descriptive Statistics of Potential Variables (N=144)

| Variable name | Sample capacity | Crest value | Least value | Average value | Standard error |
| --- | --- | --- | --- | --- | --- |
| Changes in laws and policies | 144 | 4 | 1 | 1.59 | 0.978 |
| Natural disasters | 144 | 5 | 1 | 1.84 | 1.029 |
| Changes in market demand | 144 | 5 | 1 | 2 | 0.916 |
| Changes in the economic environment | 144 | 5 | 1 | 1.951 | 1.118 |
| Manufacturing costs are too high | 144 | 5 | 1 | 2.292 | 1.083 |
| Production technology is not enough to meet the quality requirements | 144 | 4 | 1 | 2.035 | 0.904 |
| Low quality of building raw materials does not meet the requirements | 144 | 4 | 1 | 2.604 | 1.219 |
| Design pattern change | 144 | 5 | 1 | 2.049 | 1.02 |
| Whether the transportation company has a credit | 144 | 4 | 1 | 2.083 | 0.865 |
| Transportation cost of assembly parts | 144 | 4 | 1 | 1.958 | 1.09 |
| Rationality of the transportation standards | 144 | 4 | 1 | 2.306 | 0.887 |
| Project subcontracting | 144 | 5 | 1 | 2.472 | 0.931 |
| Information sharing | 144 | 5 | 1 | 1.993 | 1.226 |
| Design scheme accuracy | 144 | 5 | 1 | 2.354 | 1.162 |
| Supply chain structure | 144 | 5 | 1 | 2.917 | 1.021 |
| Strategic planning accuracy | 144 | 5 | 1 | 2.361 | 1.132 |
| Product quality | 144 | 5 | 1 | 2.021 | 0.889 |
| Warehousing expense | 144 | 5 | 1 | 2.507 | 1.194 |
| Procurement price | 144 | 5 | 1 | 2.521 | 1.165 |
| Performance capacity of the supplier | 144 | 5 | 1 | 2.389 | 1.097 |
| Poor return and maintenance ability | 144 | 5 | 1 | 2.528 | 1.14 |
| Quality of the completion acceptance is unqualified | 144 | 5 | 1 | 2.451 | 1.07 |
| Safety misadventure | 144 | 5 | 1 | 2.056 | 1.151 |
| Duration control | 144 | 4 | 1 | 1.875 | 0.93 |

Table B.2 Total variance interpretation in the questionnaire

| Total variance interpretation | | | | | | | | | |
| --- | --- | --- | --- | --- | --- | --- | --- | --- | --- |
| Initial eigenvalue | | | | Extraction sum of squares of load | | | Sum of the squares of the rotating load | | |
| Ingredient | Amount to | Variance percentage | Accumulate% | Amount to | Variance percentage | Accumulate% | Amount to | Variance percentage | Accumulate% |
| 1 | 10.310 | 42.957 | 42.957 | 10.310 | 42.957 | 42.957 | 3.429 | 14.287 | 14.287 |
| 2 | 1.895 | 7.898 | 50.855 | 1.895 | 7.898 | 50.855 | 3.319 | 13.829 | 28.115 |
| 3 | 1.861 | 7.754 | 64.642 | 1.861 | 7.754 | 58.608 | 3.253 | 13.556 | 41.671 |
| 4 | 1.448 | 6.034 | 70.269 | 1.448 | 6.034 | 64.642 | 3.121 | 13.003 | 54.675 |
| 5 | 1.351 | 5.627 | 75.230 | 1.351 | 5.627 | 70.642 | 2.719 | 11.327 | 66.002 |
| 6 | 1.191 | 4.961 | 78.447 | 1.191 | 4.961 | 75.230 | 2.215 | 9.228 | 75.230 |
| 7 | 0.772 | 3.217 | 81.265 |  |  |  |  |  |  |
| 8 | 0.676 | 2.818 | 83.265 |  |  |  |  |  |  |
| 9 | 0.583 | 2.429 | 83.694 |  |  |  |  |  |  |
| 10 | 0.497 | 2.071 | 85.765 |  |  |  |  |  |  |
| 11 | 0.434 | 1.810 | 87.575 |  |  |  |  |  |  |
| 12 | 0.405 | 1.689 | 89.265 |  |  |  |  |  |  |
| 13 | 0.371 | 1.545 | 90.810 |  |  |  |  |  |  |
| 14 | 0.367 | 1.530 | 92.340 |  |  |  |  |  |  |
| 15 | 0.301 | 1.254 | 93.595 |  |  |  |  |  |  |
| 16 | 0.271 | 1.129 | 94.724 |  |  |  |  |  |  |
| 17 | 0.229 | 0.955 | 95.678 |  |  |  |  |  |  |
| 18 | 0.196 | 0.816 | 96.494 |  |  |  |  |  |  |
| 19 | 0.177 | 0.736 | 97.230 |  |  |  |  |  |  |
| 20 | 0.155 | 0.647 | 97.877 |  |  |  |  |  |  |
| 21 | 0.148 | 0.615 | 98.492 |  |  |  |  |  |  |
| 22 | 0.145 | 0.603 | 99.095 |  |  |  |  |  |  |
| 23 | 0.120 | 0.501 | 99.596 |  |  |  |  |  |  |
| 24 | 0.097 | 0.404 | 100.00 |  |  |  |  |  |  |

**Appendix C**

Table C.1 Variable values and their expressions

| Type of variable | Name | Take value and equation |
| --- | --- | --- |
| Constant | Transportation company credit | 0.03572 |
|  | Component transportation cost | 0.03572 |
|  | Reasonableness of transportation standards | 0.03714 |
|  | Duration control | 0.02879 |
|  | Change of design drawings | 0.03623 |
|  | Warehousing expense | 0.04646 |
|  | Strategic planning accuracy | 0.04752 |
|  | Procurement price | 0.04489 |
|  | Product quality | 0.03714 |
|  | Design scheme accuracy | 0.04593 |
|  | Project subcontracting | 0.03908 |
|  | Supply chain structure | 0.03292 |
|  | Information sharing | 0.03292 |
|  | Production cost is too high | 0.03696 |
|  | Changes in the economic environment | 0.04034 |
|  | Low quality of raw materials | 0.03969 |
|  | Low production technology | 0.03696 |
|  | Supplier's performance capacity | 0.05319 |
|  | Poor return and maintenance ability | 0.04813 |
|  | Unsatisfactory quality of acceptance | 0.04768 |
|  | Natural disasters | 0.04306 |
|  | Changes in market demand | 0.05498 |
|  | Changes in laws and policies | 0.05096 |
| Horizontal variable | Transportation process | INTEG (variation of transport process influencing factors, 0.197) |
|  | Manufacturing process | INTEG (variation of manufacturing process influencing factors, 0.162) |
|  | Purchasing process | INTEG (variation of purchasing process influencing factors, 0.176) |
|  | Planning process | INTEG (variation of planning process influencing factors, 0.167) |
|  | Exotic environment | INTEG (variation of external environment influencing factors, 0.149) |
|  | Delivery and use | INTEG (variation of transport process influencing factors, 0.149) |
| Rate variable | Variation of manufacturing process influencing factors | Low production technology * 0.03696 + Production cost is too high * 0.03969 + Low quality of raw materials * 0.03696 + Economic environment change * 0.04034 |
|  | Variation of purchasing process influencing factors | Product quality * 0.03714 + Storage cost * 0.04646 + Strategic planning accuracy * 0.04752 + Purchase price * 0.04489 |
|  | Variation of planning process influencing factors | Supply chain structure * 0.02308 + Information sharing * 0.04292 + Project subcontracting * 0.03908 + Design scheme accuracy * 0.04593 |
|  | Variation of transport process influencing factors | Transportation company credit * 0.03572 +Component transportation cost * 0.03572 + Reasonableness of transportation standards * 0.03714 + Duration control * 0.02879 + Change of design drawings* 0.03623 |
|  | Variation of external environment influencing factors | Changes in market demand * 0.05498 + Changes in laws and policies * 0.05096 +Natural disasters * 0.04306 |
|  | Variation of delivery and use influencing factors | Supplier's performance capacity * 0.05319 + Unsatisfactory quality of acceptance * 0.04768 + Poor maintenance and return ability * 0.04813 |

**Legends**

**Table A.1:** Questionnaire responses displayed in the table capture the perceived importance of various factors impacting supply chain resilience in the prefabricated building industry. Respondents rated each item based on a scale ranging from 'Have nothing to do with' to 'Very important', providing insights into the significance attributed to each factor, such as changes in laws and policies, natural disasters, market demand, and transportation costs.

**Table B.1:** Descriptive Statistics of Potential Variables (N=144) provides a snapshot of the data collected from respondents, with measurements for various factors that could influence the supply chain resilience. It includes sample size, the highest and lowest values observed (Crest and Least value), average scores, and the standard error for each variable.

**Table B.1:** Total variance interpretation in the questionnaire displays the results from a factor analysis, detailing the initial eigenvalues, the percentage of variance each component explains, and the cumulative percentage. This helps to understand the data structure and the importance of each factor in explaining the variability in the questionnaire responses.

**Table C.1:** Variable values and their expressions in the study are presented in this table, which lists the assigned value for each constant and rate variable affecting the resilience of the prefabricated building supply chain. Constants represent fixed contributions to resilience, while rate variables are multiplied by specific influencing factors, reflecting their proportional impact within the model's calculations.
